# Mucin‐Like Glycoproteins Modulate Interfacial Properties of a Mimetic Ocular Epithelial Surface

**DOI:** 10.1002/advs.202100841

**Published:** 2021-06-29

**Authors:** Chunzi Liu, Amy C. Madl, Daniel Cirera‐Salinas, Wolfgang Kress, Frank Straube, David Myung, Gerald G. Fuller

**Affiliations:** ^1^ Department of Chemical Engineering Stanford University Stanford CA 94305 USA; ^2^ Global Drug Development Biopharmaceutical Process & Product Development Novartis Pharma Basel AG 4002 Switzerland; ^3^ Department of Ophthalmology Stanford University Stanford CA 94305 USA

**Keywords:** contact angle hysteresis, dry eye disease, interfacial properties, lubricin, muco‐adhesion, ocular surfaces

## Abstract

Dry eye disease (DED) has high personal and societal costs, but its pathology remains elusive due to intertwined biophysical and biochemical processes at the ocular surface. Specifically, mucin deficiency is reported in a subset of DED patients, but its effects on ocular interfacial properties remain unclear. Herein a novel in vitro mucin‐deficient mimetic ocular surface (Mu‐DeMOS) with a controllable amount of membrane‐tethered mucin molecules is developed to represent the diseased ocular surfaces. Contact angle goniometry on mimetic ocular surfaces reveals that high surface roughness, but not the presence of hydrophilic mucin molecules, delivers constant hydration over native ocular surface epithelia. Live‐cell rheometry confirms that the presence of mucin‐like glycoproteins on ocular epithelial cells reduces shear adhesive strength at cellular interfaces. Together, optimal surface roughness and surface chemistry facilitate sustainable lubrication for healthy ocular surfaces, while an imbalance between them contributes to lubrication‐related dysfunction at diseased ocular epithelial surfaces. Furthermore, the restoration of low adhesive strength at Mu‐DeMOS interfaces through a mucin‐like glycoprotein, recombinant human lubricin, suggests that increased frictional damage at mucin‐deficient cellular surfaces may be reversible. More broadly, these results demonstrate that Mu‐DeMOS is a promising platform for drug screening assays and fundamental studies on ocular physiology.

## Graphical abstract

Lubrication dysfunction associated with dry eye disease lacks an effective treatment. This study reports an in vitro cell model mimicking ocular surfaces of mucin‐deficient dry‐eye patients. The model reveals that both surface roughness and surface chemistry are crucial to an effective lubrication. In addition, a recombinant glycoprotein can reverse the increased friction at ocular epithelial interface induced by mucin deficiency.

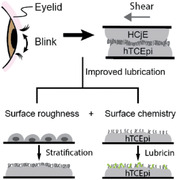

## 1 Introduction

Dry eye disease (DED) affects hundreds of millions of people worldwide and is one of the most common reasons patients visit eye care practitioners.^[^ ^1^ ^]^ DED is characterized by disruptions in tear film homeostasis that result in altered mechanical interactions between the eyelid and the globe during spontaneous blinks.^[^ ^2^, ^3^, ^4^ ^]^ In a subset of DED patients, reduced biosynthesis or loss of functional mucins has been observed and hypothesized to cause defective lubrication that drives pathophysiology.^[^ ^1^, ^3^, ^4^, ^5^ ^]^ However, the effects of mucin deficiency on live cell interfacial properties at the apical membrane has not been directly quantified due to a lack of a suitable model system and measurement techniques.

Effective lubrication is critical for maintaining homeostasis at biological surfaces that are exposed to constant external stresses, such as ocular and cartilage surfaces. Biolubrication is a function of tissues’ heterogeneous molecular composition, morphology, and surrounding aqueous environments.^[^ ^6^, ^7^ ^]^ Although mechanisms such as boundary lubrication and elasto‐hydrodynamic lubrication have been proposed to explain the lubricious nature of biological tissues, much remains unknown about differential biolubrication mechanisms in healthy and diseased tissues.^[^ ^5^, ^8^, ^9^ ^]^ Of specific relevance to treatments for mucin‐deficient DED, the role of membrane‐tethered ocular surface mucins in friction reduction and tear film maintenance remains poorly understood, despite previous studies examining the effects of purified mucin conformation, intermolecular force, and adsorption mechanics on biomimetic surfaces.^[^ ^10^, ^11^, ^12^ ^]^

Mucins are glycoproteins characterized by central tandem repeats of amino acids which are rich in serine, threonine, and proline, where the serine and threonine residues serve as O‐glycosylation sites.^[^ ^13^ ^]^ In healthy individuals, the ocular surface is covered by a concentrated layer of membrane‐tethered mucin molecules such as mucin‐1 (MUC1), mucin‐4, and mucin‐16, along with a shed and secreted mucins that form the muco‐aqueous layer of the eye's protective tear film.^[^ ^14^ ^]^ These large, negatively charged glycoproteins are believed to be involved in hydrating, protecting, and lubricating the ocular surface, while also retaining antimicrobial proteins and resisting particulate and pathogenic adhesion.^[^ ^14^, ^15^ ^]^ In addition to ocular surface mucins, the amphiphilic, mucin‐like glycoprotein lubricin is transcribed and translated at low levels by ocular surface epithelial cells and appears to function as a boundary lubricant.^[^ ^16^, ^17^ ^]^

To better understand how alterations in the presentation of mucin‐like glycoproteins contribute to vision‐threatening pathology in DED patients, we developed in vitro model ocular surface using immortalized human conjunctival and corneal epithelial cell lines. Previously, model systems reliant on donor tissue or small animals have been employed. However, the natural mucin‐rich glycocalyx present on apical membranes of donor tissue samples is easily damaged during transport.^[^ ^18^ ^]^ Moreover, interspecies physiology differences limit insights that can be drawn about the human ocular surface from small animal models.^[^ ^19^, ^20^ ^]^ Accordingly, alternative model systems with controlled mucin presentation are needed to study the interfacial properties of membrane‐tethered mucins. Here we applied a mucin‐specific protease, secreted protease of C‐1 esterase inhibitor (StcE), in the in vitro cellular model to mimic the mucin‐deficient ocular surface (Mu‐DeMOS) observed in some DED patients.^[^ ^21^, ^22^, ^23^ ^]^

By employing Mu‐DeMOS in contact angle (CA) goniometer and live cell rheology experiments, we quantified how partial removal of membrane‐tethered mucins from the apical cell membranes affects interfacial and adhesive properties, including cell surface hydrophilicity, fluid retention force of cell surfaces, and shear adhesive strength at cell‐cell interfaces. Specifically, the results demonstrated that membrane‐tethered mucins are not a direct driving force behind cell surface hydrophilicity or fluid retention on model ocular surfaces. However, partial removal of membrane‐tethered mucins increased shear adhesive strength at cell‐cell interfaces, suggesting that membrane‐tethered mucins are crucial in reducing frictional damages during blink cycles. We uncovered that high surface roughness of epithelial cell layers correlates with high contact angle hysteresis (CAH), which potentially facilitates fluid film retention in a healthy ocular surface. In addition, supplementation with the recombinant human lubricin restored the low adhesive strength at the model mucin‐deficient conjunctival/corneal epithelia. Together, these results highlight the potential of our biomimetic ocular surface model system as a modular system for future studies on symptoms and treatments of mucin‐deficient DED and suggest a framework to study lubrication mechanisms of other biological surfaces.

## 2 Results

### 2.1 Development and Characterization of Mucin‐Deficient Mimetic Ocular Surfaces (Mu‐DeMOS)

**Figure 1:**
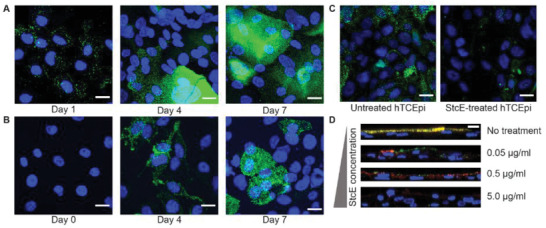
Immunofluorescent imaging of the differentiation and stratification process of hTCEpi and HCjE cells. Representative images of differentiation process of A) hTCEpi cells post medium change day 1, 4, and 7; B) HCjE cells post medium change day 0, 4, and 7. Scale bar: 20 µm. C) StcE treatment successfully removed MUC1 from hTCEpi cell surface. Scale bar: 20 µm. Green: MUC1; blue: nucleus. D) Glycocalyx was removed by StcE in a dose‐dependent fashion. Scale bar: 20 µm. Red: MUC1; green: jacalin‐FITC; blue: nucleus.

Immortalized human corneal (hTCEpi) and conjunctival (HCjE) epithelial cells capable of reproducibly differentiating into stratified cellular layers that express membrane‐tethered mucins were employed to model healthy corneal and inner eyelid epithelial surfaces.^[^ ^24^, ^25^, ^26^, ^27^ ^]^ To mimic the stratified structures of the ocular epithelia, we induced hTCEpi and HCjE differentiation by culturing confluent monolayers in high calcium, serum‐containing stratification medium as previously reported (Experimental Section).^[^ ^24^, ^25^ ^]^ As anticipated, substantial changes in mucin expression level and cell surface morphology were observed for both hTCEpi and HCjE cell layers over the course of seven days in stratification medium. Apical membrane presentation of the membrane‐tethered mucin mucin‐1 (MUC1) was verified by immunofluorescence imaging (**Figure** **1**A,B). After 7 days, differentiated hTCEpi cells exhibited a continuous, MUC1‐rich apical surface, while differentiated HCjE cells presented apical MUC1 islands along with some subapical mucin expression, consistent with previously reported differences in mucin presentation between immortalized corneal and conjunctival epithelial cells.^[^ ^25^ ^]^

In conjunction with increased MUC1 expression, the morphology of the immortalized human corneal epithelial cells gradually changed from a cobblestone topology to a flattened topology during the differentiation process. Localization of the tight junction marker zonula occludens‐1 (ZO‐1) shifted from the nucleus to cell‐cell junctions near the apical surface over 7 days (Figure S1, Supporting Information), recapitulating the apical features of the mature human corneal epithelium.

To mimic the altered mucin expression observed in many DED patients, we developed a Mu‐DeMOS model by treating differentiated hTCEpi and HCjE cells with recombinant StcE. StcE is a bacterial enzyme purified from *Escherichia coli* that specifically cleaves *α*‐O‐glycan‐containing substrates with a peptide consensus sequence S/T*‐X‐S/T, where X is any amino acid and the asterisk represents glycosylation, common in mucin and mucin‐like glycoproteins. The enzymatic activity of StcE has been verified in vitro for BT‐20, HeLa, and MDA‐MB‐453 cells under cell culture conditions.^[^ ^21^ ^]^ StcE activity against membrane‐tethered mucins on model ocular surface cell layers was confirmed by lectin fluorescent labeling against O‐glycans and immunofluorescence staining for MUC1 (Figure 1C,D). Overnight incubation with 5 µg mL^−1^ StcE substantially depleted the MUC1 rich layer on the differentiated hTCEpi cell surface (Figure 1C), and StcE treatment for three hours resulted in a dose‐dependent decrease in cell surface glycoproteins (Figure 1D). Overall, StcE treatment on differentiated hTCEpi and HCjE generated cellular surfaces with a controllable amount of endogenous membrane‐tethered mucins.

### 2.2 Surface Roughness Increased the Contact Angle Hysteresis of the Model Corneal Epithelium

**Figure 2:**
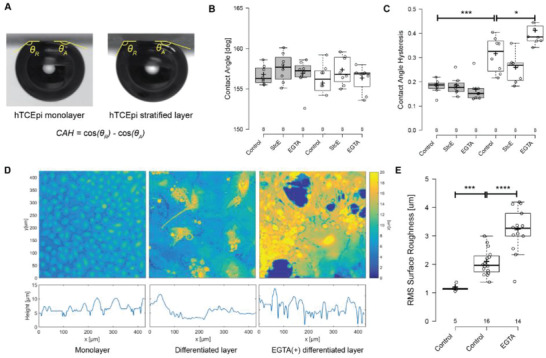
Surface roughness increased CAH of hTCEpi cell surfaces. A) Representative images for the advancing and receding CA measurements on hTCEpi cell surfaces. Air bubble size: 5 µL. B) CA and C) CAH for hTCEpi monolayers (gray) and stratified layers (white) under control, StcE‐treated, and EGTA‐treated conditions. D) Representative height maps and cross‐sectional height reconstructed from fluorescence images and E) root‐mean square surface roughness quantified from the height maps (see Experimental Section) for hTCEpi monolayers, stratified layers, and stratified layers under EGTA treatment. Box plot: center lines show the medians; box limits indicate the 25th and 75th percentiles as determined by R software; whiskers extend 1.5 times the interquartile range from the 25th and 75th percentiles; crosses represent sample means; data points are plotted as open circles; sample numbers are shown above condition name. Student t‐test was used to report the two‐tail *p*‐value. *: *p*<0.05; ***: *p*<0.001; ****: *p*<0.0001.

To determine how differentiation and membrane‐tethered mucin expression affect the surface properties of the model human corneal epithelium, we measured the CA and CAH of differentiated hTCEpi layers using the captive bubble method (**Figure** **2**A). We define CAH as the difference in cosines of the advancing and receding CAs when bubble escapes cell surfaces. Before inducing differentiation, the CA of hTCEpi monolayers was 156.7° ± 0.4° (mean ± S.E.); this value did not significantly change following differentiation (*p* = 0.361) or post‐StcE treatment (*p* = 0.209) to induce mucin deficiency (Figure 2B). However, the CAH increased from 0.182 ± 0.010 for the monolayers to 0.312 ± 0.025 for differentiated hTCEpi cells (Figure 2C, *p* = 2.9 × 10^−4^), consistent with previous reports.^[^ ^24^ ^]^ Intriguingly, the observed CAH was not significantly different between control and mucin‐deficient differentiated hTCEpi cell layers (Figure 2C, *p* = 0.104), suggesting that the observed increase in CAH following differentiation is not attributable to the expression of membrane‐tethered mucins during the differentiation process.

As surface heterogeneity influences CAH,^[^ ^28^ ^]^ we hypothesized that changes in cell surface morphology during the differentiation process may contribute to the observed increase in CAH. To quantify changes in surface heterogeneity following differentiation, we fluorescently labeled the lipid bilayers of hTCEpi cell layers and generated cell surface height maps. As shown in Figure 2D, the surfaces of confluent hTCEpi monolayers exhibited a cobblestone morphology while the differentiated hTCEpi layers exhibited a flattened morphology on the same length scale. The root‐mean‐square surface roughness was used to quantify the amplitude of the surface irregularities (Experimental Section). As shown in Figure 2E, differentiated hTCEpi cell layers exhibited a root‐mean‐square surface roughness of 2.06 ± 0.11 µm, greater than that of confluent monolayers at 1.17 ± 0.05 µm (*p* = 3.3 × 10^−4^).

To test the correlation between cell surface roughness and CAH, we perturbed the cell surface morphology by exposing differentiated hTCEpi layers to a stratification medium supplemented by 1 mm of a calcium chelator, ethylene glycol‐bis(*β*‐aminoethyl ether)‐*N*,*N*,*N*′,*N*′‐tetra‐acetic acid (EGTA). Calcium ions are required for the formation and maintenance of cell‐cell adhesion complexes, and the disruption of cell‐cell contacts has been reported to significantly alter epithelial surface morphology.^[^ ^29^ ^]^ In contrast to the stratification medium prepared using DMEM/F‐12 (1.05 mm Ca^2+^ according to the manufacturer), the calcium content of EGTA‐supplemented DMEM/F‐12 was estimated to be approximately 60 µm using the CHELATOR algorithm.^[^ ^30^ ^]^ The root‐mean‐square surface roughness of differentiated hTCEpi cell layers increased to 3.27 ± 0.20 µm after EGTA treatment (Figure 2E, *p* = 2.3 × 10^−5^). The CA of hTCEpi monolayers and stratified layers did not significantly change upon the EGTA treatment (Figure 2B), indicating that the hydrophilicity of the cell surface was not affected by cell‐cell contact disruption. However, the observed CAH for differentiated hTCEpi cells increased from 0.312 ± 0.025 to 0.408 ± 0.024 after EGTA treatment (Figure 2C, *p* = 0.015), in accordance with the increase in the RMS surface roughness (Figure 2E), indicating that surface roughness positively correlates with CAH.

### 2.3 Mucin Deficiency Increases the Adhesive Strength at Mu‐DeMOS Cell‐Cell Interfaces

A well‐lubricated ocular surface enables free eyelid motion, with mucins facilitating hydrophilic brush‐to‐brush lubrication due to their high hydration extents and O‐glycan related repulsive forces.^[^ ^31^, ^32^ ^]^ In lubrication‐related ocular disorders, the mechanical attributes of the ocular surface responsible for free eyelid motion—tear film stability, surface quality, interface load, and eyelid dynamics—are perturbed.^[^ ^31^ ^]^ To specifically interrogate the effects of membrane‐tethered mucins on the biolubrication functions of model healthy and diseased ocular tissue, we conducted stress relaxation experiments on stratified hTCEpi and HCjE cell layers with a custom‐built live cell rheometer (LCR) that was developed by the Fuller laboratory.^[^ ^33^, ^34^, ^35^ ^]^ Cells were subjected to a step‐shear deformation, and the subsequent force response was monitored. The apparent modulus of the cells, defined as the shear stress over the shear strain, was evaluated as a function of time (Experimental Section).

**Figure 3:**
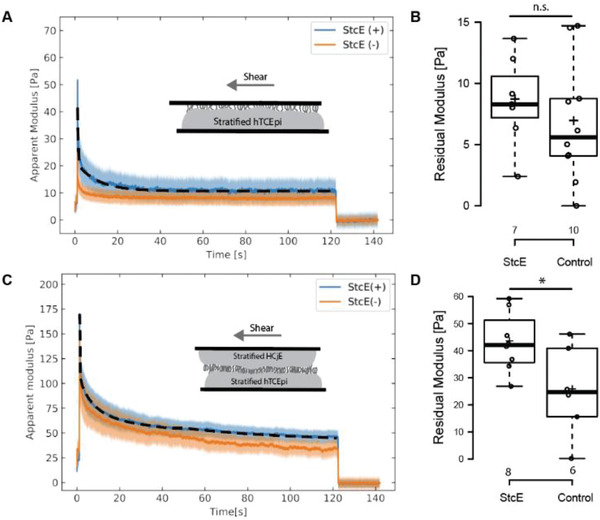
Stress relaxation behaviors of differentiated hTCEpi and HCjE cell layers. A,C) Averaged stress relaxation curves and B,D) residual moduli from LCR on differentiated hTCEpi against collagen‐coated glass surfaces and differentiated hTCEpi/HCjE interfaces under control and StcE‐treated conditions. Stress relaxation curves: shaded areas represent standard errors; black dashed lines are fitted two‐component exponential curves. Student t‐test was used to report the two‐tail *p*‐value. *: *p*<0.05.

To investigate the rheological properties of the corneal surface epithelium, we first evaluated the relaxation behavior of differentiated hTCEpi cell layers in contact with collagen‐coated glass (**Figure** **3**A). Differentiated hTCEpi cell layers can dissipate stress by rearranging cellular components, such as cytoskeletal molecules, or relaxing intermolecular interactions at the cell‐collagen interface. As shown in Figure 3A, the apparent modulus of the differentiated hTCEpi cell layers in contact with collagen‐coated glass peaked immediately following the imposed step strain and relaxed to a stable plateau (the plateau modulus) at a long‐time scale (> 10 s), consistent with previous rheological characterizations of hTCEpi monolayers and bladder epithelial cells.^[^ ^33^, ^34^ ^]^ While the short‐time scale relaxation behavior reflects both rearrangements of internal cellular structures and intermolecular bonds at the interface, the plateau modulus results from interactions at the cell/collagen interface that continue to resist the imposed strain at long time scales. Therefore, we used the plateau modulus as an indication of the adhesive strength of the cell layer.

The plateau modulus, *G*_0_, was obtained by fitting the relaxation curve to a double exponential decay function of the following form,

(1) $$G\;\left(t\right)=G_{1}\;\exp\left(-\frac{t}{\tau_{1}}\right)+G_{2}\exp\left(-\frac{t}{\tau_{2}}\right)+G_{0}$$

where *G*_1_, *G*_2_, *τ*_1_, *τ*_2_, and *G*_0_ are fitting parameters. We chose a double exponential decay function due to the separate time scales of the two proposed relaxation mechanisms, which were not captured by a single exponential decay function. An improved fitting of short‐time relaxation behaviors by the double exponential decay function is shown in Figure S2, Supporting Information.

To mimic the ocular surface environment during blink cycles and assess adhesions between the corneal and the conjunctival epithelial cells lining the inner eyelid, we measured the relaxation behavior of a cell‐on‐cell geometry. To model the corneal surface and the inner eyelid, differentiated hTCEpi and HCjE cell layers were cultured on top and bottom plates, respectively (Figure 3B). The height of the cell layers was measured with fluorescent confocal imaging (Figure S3, Supporting Information). Prior to the step‐strain experiment, the differentiated cell surfaces were contacted for a minimum of two hours to enable adhesion formation, which was previously shown as sufficient to equilibrate the adhesive strength at the cellular interface.^[^ ^35^ ^]^ Like differentiated hTCEpi cells contacting collagen‐coated glass, the cell‐on‐cell model exhibited relaxation behavior resembling that of a viscoelastic soft solid material. However, the magnitude of the relaxation modulus in the cell‐on‐cell model was fivefold higher than that observed for stratified hTCEpi cell layers in contact with collagen‐coated glass. The higher plateau modulus may reflect improved interfacial contact in the cell‐on‐cell model, as well as stronger adhesive interactions between the two cell layers relative to cell layers contacting collagen‐coated glass (Discussion).

To investigate the contributions of membrane‐tethered mucin molecules to the adhesive strength at the cell‐cell interface, we performed stress relaxation experiments on Mu‐DeMOS. Induced mucin deficiency at the apical surfaces increased the plateau modulus of the cell‐on‐cell model from 25.4 ± 6.8 Pa (mean ± S.D.) to 43.0 ± 3.9 Pa (*p* = 0.017), suggesting that mucin‐deficient ocular epithelial cells formed stronger interfacial adhesions. The increase in modulus was less significant than that observed for differentiated hTCEpi cell layers against collagen‐coated surfaces following StcE treatment.

The relaxation behaviors observed using LCR represent a complex superposition of the adhesive and mechanical properties of the cell layers. To better understand the contributions of cell mechanics to the observed relaxation behavior, we measured the single‐cell Young's modulus by indentation with an atomic force microscope (AFM). As shown in Figure S4, Supporting Information, Young's modulus of a single cell in differentiated hTCEpi cell layers was 4.93 ± 0.34 kPa and did not change upon StcE treatment (5.41 ± 0.51 kPa, *p* = 0.234), indicating that differential adhesive properties induced by the lack of mucin molecules at the cell‐cell interface, rather than potential differences in cell mechanical property between the control and StcE‐treated cell layers, drove the observed changes in relaxation behavior.

### 2.4 Human Recombinant Lubricin Adsorption Restored Lubrication Function on Mu‐DeMOS

To further understand the role of mucin‐like glycoproteins in maintaining a lubricious ocular surface, here we investigated the contribution of lubricin, a glycoprotein first characterized in the articular cartilage,^[^ ^36^, ^37^ ^]^ to the adhesive properties at Mu‐DeMOS interfaces.

Endogenous lubricin is believed to function as a boundary lubricant at biological surfaces, including the ocular surface, and exogenous recombinant human lubricin (rh‐lubricin) molecules have been hypothesized to restore glycocalyx in DED patients with deficient or dysfunctional ocular surface mucins.^[^ ^17^ ^]^ Here we investigated the adsorption and lubrication properties of rh‐lubricin molecules on model healthy and mucin‐deficient cellular surfaces to better understand how rh‐lubricin interacts with the ocular surface glycocalyx. Purified rh‐lubricin exhibited a molecular weight around 460 kDa (Figure S5A, Supporting Information), similar in size to endogenous lubricin and recombinant human lubricin generated by other laboratories.^[^ ^16^, ^38^ ^]^ Additionally, DLS measurement showed that the hydrodynamic diameter of purified rh‐lubricin was 65.4 ± 1.1 nm (Figure S5C, Supporting Information), consistent with previous reports.^[^ ^39^ ^]^ A lectin blot using FITC‐Jacalin also confirmed the presence of O‐glycans on purified rh‐lubricin (Figure S5B, Supporting Information). Moreover, purified rh‐lubricin possesses sialic acid residues, as shown by the downward shift in the molecular weight following sialidase treatment. Additionally, StcE treatment at 37 °C for three hours shifted the molecular weight of purified rh‐lubricin to a faint 60–70 kDa band on the Western blot (Figure S5A, Supporting Information), indicating rh‐lubricin possesses accessible, glycosylated sequences in its protein backbone. To avoid StcE digestion of rh‐lubricin, mucin‐deficient cell surfaces were extensively washed prior to incubation in lubricin‐supplemented medium to remove adsorbed enzymes.

**Figure 4:**
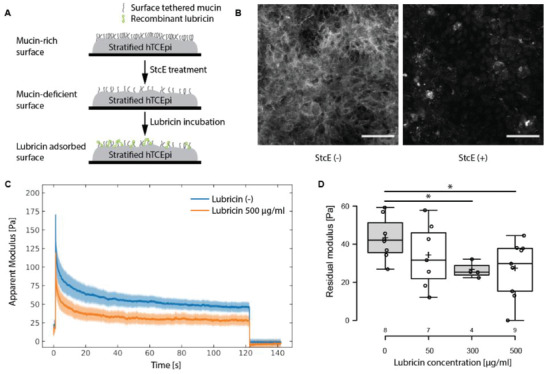
Lubricin adsorption at mucin‐deficient cell surfaces. A) An illustration of adsorption assay. B) Live cell imaging revealed the adsorption behavior of Cy5‐conjugated recombinant lubricin molecules on stratified hTCEpi surfaces. Cy5‐conjugated rh‐lubricin was homogeneously adsorbed on differentiated hTCEpi cell surfaces, while formed clusters post‐StcE treatment. Scale bar: 100 µm. C) Stress relaxation curves from LCR on StcE‐treated hTCEpi/HCjE interface with the addition of lubricin. D) Residual moduli extracted from the stress relaxation curves at different lubricin concentrations. Student t‐test was used to report the two‐tail *p*‐value. *: *p*<0.05.

To interrogate the adsorption behavior of rh‐lubricin molecules on model corneal epithelial surface, we conjugated rh‐lubricin with Cy5 fluorophores and detected its presence on differentiated hTCEpi cell surfaces after a two‐hour incubation at 50 µg mL^−1^. A schematic of the assay is described in **Figure** **4**A. A substantial amount of rh‐lubricin is adsorbed on the differentiated hTCEpi cell surface relative to Cy5‐conjugated streptavidin molecules (Figure 4B and Figure S6A, Supporting Information). Post‐incubation immunofluorescence also confirmed rh‐lubricin adsorption (Figure S6B, Supporting Information). A lower yet significant adsorption level was observed on mucin‐deficient hTCEpi cell surfaces (Figure 4B and Figure S7A,B, Supporting Information), and the adsorbed rh‐lubricin molecules formed clusters instead of a homogenous adsorbed layer as seen on the non‐StcE treated cell surfaces. To verify that the signal on StcE‐treated cell was not caused by non‐specific interactions between Cy5 and cell surfaces, we repeated the adsorption experiment with un‐modified rh‐lubricin and verified its presence on StcE‐treated cell surfaces post‐incubation with immunofluorescent imaging (Figure S7C,D, Supporting Information). The twofold increase in the mean fluorescent intensity after adsorption confirmed a specific interaction between rh‐lubricin and StcE‐treated cell surfaces. Lubricin is known to associate with cell surface glycoproteins and various extracellular matrix components, including collagen, fibronectin, and hyaluronate.^[^ ^17^ ^]^ The homogenous presence of rh‐lubricin on mucin‐rich cell surfaces was consistent with its reported tendency to associate with glycosylated proteins, while the cluster formation on the mucin‐deficient cell surface may reflect rh‐lubricin sequestration with remaining patches of membrane‐tethered mucins (Discussion).

The lubrication properties of rh‐lubricin on model ocular surfaces during simulated blink cycles were assessed by applying a step strain to the mucin‐deficient cell‐on‐cell model in the presence of rh‐lubricin supplemented medium. As shown in Figure 4C, the presence of rh‐lubricin in the surrounding medium shifted the relaxation curve in the mucin‐deficient cell‐on‐cell model to a lower modulus value and decreased its plateau modulus from 43.0 ± 3.9 Pa in unsupplemented medium to 26.3 ± 2.1 Pa in medium containing 300 µg mL^−1^ rh‐lubricin (*p* = 0.009) and 27.0 ± 4.9 Pa in medium containing 500 µg mL^−1^ rh‐lubricin (*p* = 0.012). This behavior does not appear to be attributable to nonspecific protein interactions as the model interface as bovine serum albumin (BSA) supplementation did not alter the plateau modulus in the mucin‐deficient cell‐on‐cell model (Figure S8D, Supporting Information). Supplementation with rh‐lubricin had less impact at the healthy (non‐StcE treated) cell‐on‐cell interface, with the plateau modulus exhibiting a minor increase at an rh‐lubricin concentration of 500 µg mL^−1^, possibly due to overcrowding of glycoproteins on the apical cell surfaces (Figure S8A, Supporting Information). A slight increase in the plateau modulus was also seen at a BSA concentration of 300 µg mL^−1^ in the non‐StcE treated cell‐on‐cell model (Figure S8C, Supporting Information). In conclusion, supplementation with rh‐lubricin in the surrounding medium specifically reduced the stress relaxation modulus at model mucin‐deficient ocular interfaces in a dose‐dependent fashion.

## 3 Discussion

Interfacial interactions are paramount for the physiological functions of the ocular surface, including hydration maintenance and healthy blink cycles. To investigate lubrication mechanisms at the ocular surface, we employed model healthy and mucin‐deficient cell layers in CAH measurements and live cell rheology experiments. Induced mucin deficiency increased friction at model cell‐on‐cell interfaces, consistent with the frictional damage and symptoms of discomfort experienced by patients suffering from dye eye disease (DED). In contrast, CA and CAH measurements were not significantly affected by partial removal of membrane‐tethered mucins, suggesting that mucin dysfunction may not contribute directly to the ocular surface hydration issues observed in DED patients. In addition, increased epithelial cell surface roughness correlated with increased CAH, indicating improved retentive force on the model tear film. The results indicate that both surface roughness and surface chemistry are critical for consistent lubrication at healthy ocular surfaces.

Maintaining the hydrophilicity of the corneal epithelium is important for tear film stability and homeostasis, both of which are disrupted in DED. Epithelial cell surfaces are generally hydrophilic due to the presence of membrane‐tethered glycosylated proteins.^[^ ^3^ ^]^ To investigate the specific contributions of membrane‐tethered mucins to the hydrophilicity of the ocular surface, we measured the CA for differentiated hTCEpi cell layers using the captive bubble method. As the CA at a three‐phase boundary reflects the wettability at liquid/solid interfaces, the high CA values of differentiated hTCEpi cell layers against air indicated the cell surface was highly hydrophilic. The CA of differentiated hTCEpi cell layers remained stable following partial removal of membrane‐tethered mucins (Figure 2B), suggesting that other glycosylated proteins on the cell surface are sufficient to maintain cell surface hydrophilicity. Accordingly, the presence of a dysfunctional mucin layer may not be a direct contributor to the reduction in aqueous tear volume observed in many patients suffering from DED.

In addition to cell surface hydrophilicity, resistance against constant gravity draining is required to maintain tear film stability in human eyes. CAH is a measure of the energy dissipation necessary to initiate the flow of a droplet on a surface, with high CAH values reflecting high shear adhesive strength at the solid/liquid phase according to Furmidge relation.^[^ ^40^, ^41^ ^]^ Typically, when a solid surface favors the liquid phase, a high CA and low CAH are observed using the captive bubble method, consistent with the behavior of hTCEpi monolayers. Surprisingly, we observed high CAH on differentiated hTCEpi cell layers (Figure 2C) despite their high CA, a property that potentially facilitates tear film retention over the corneal epithelium. This property was not significantly affected by partial removal of membrane‐tethered mucins, indicating that other aspects of cell surface chemistry and morphology may be responsible for the unique stability of the healthy tear film.

CAH originates from the “pinning” effect of the surface irregularities, which can be either a chemical defect, such as a superhydrophobic point defect, or a physical defect, such as a protrusion from the surface.^[^ ^42^ ^]^ To evaluate how cell surface roughness affects the observed CAH behavior, an increase in the RMS surface roughness was induced using EGTA treatment, which resulted in increased CAH on differentiated corneal epithelial cell layers (Figure 2C–E). In addition, a low surface roughness in hTCEpi monolayers corresponded with a low CAH. The correlation between cell surface roughness and the observed CAH suggests that physical defects may be sufficient to induce a change in the ability of the cell surface to retain the liquid phase, which might be a mechanism employed by healthy ocular surfaces to maintain constant hydration.

While strong shear adhesive interactions between the ocular surface epithelia and the tear film are highly desirable, adhesions between the palpebral conjunctival cells lining the eyelid and the ocular surface can result in structural damage and symptoms of discomfort. In fact, low shear adhesive strength at the corneal‐conjunctival epithelial cell interface reduces wear on the ocular surface and facilitates healthy blink cycles. Because CAH does not provide a direct measurement of adhesive strength at cell‐cell interfaces, the role of cell surface chemistry, specifically the presence of membrane‐tethered mucins, on the shear adhesive strength of ocular epithelial cells was further explored using an LCR model.

As shown by the stress relaxation curves obtained using LCR, the peak and residual moduli increased at mucin‐deficient cell‐cell interfaces (Figure 3A), indicating stronger intermolecular interactions occur at the mucin‐deficient cellular interface. The peak and residual moduli have the same molecular origin as the equilibrium adhesion energy (interfacial tension), reflecting the strength and number of intermolecular bonds formed at the interface. Apical membrane adhesion at the cell‐cell interface is primarily mediated by non‐specific interactions, such as the entanglements of the macromolecules present on the cell surfaces. Negatively charged mucin molecules exhibit low entanglement with other macromolecules due to charge repulsion associated with their dense O‐glycan brush structures. In addition, mucin molecules, owing to their large hydrodynamic radii, can shield smaller surface proteins from interacting with other macromolecules in the microenvironment. Therefore, mucin molecules may function as a physical spacer at cell‐cell or cell‐material interfaces, reducing the probability of intermolecular bond formation between cell surface proteins. The increased peak and plateau moduli observed following StcE treatment support the hypothesis that membrane‐tethered mucins weaken intermolecular bond formation at the ocular surface, reducing friction during blinks.

Along with shed and secreted mucins, the mucin‐like glycoprotein lubricin has been detected in human tear samples.^[^ ^16^ ^]^ Illustratively, lubricin knockout (Prg4‐/‐) mice exhibited increased corneal damage relative to their wild‐type littermates.^[^ ^16^ ^]^ To better understand how exogenous lubricin interacts with healthy and mucin‐deficient ocular surface tissue, fluorescently labeled rh‐lubricin was incubated with model control and mucin‐deficient cell surfaces. Cy5‐modified rh‐lubricin readily adsorbed onto differentiated corneal epithelial surfaces, with rh‐lubricin clusters observed on mucin‐deficient cell surfaces (Figure 4B). Cluster formation on hTCEpi cell surfaces may result from a nucleation‐and‐growth mechanism, in which rh‐lubricin association with glycosylated membrane proteins present on the cell surface initiates nucleation. Moreover, the presence of rh‐lubricin in the surrounding medium at 300 and 500 ug mL^−1^ decreased the plateau modulus at the mucin‐deficient cell‐cell interface (Figure 4C,D), improving lubrication and attenuating the increase in friction associated with loss of membrane‐tethered mucins. The effective concentration range in our in vitro study coincides with a recent clinical trial in which eye drops containing recombinant human lubricin (rh‐lubricin) at 150 ug mL^−1^ produced significant improvements in signs and symptoms of DED.^[^ ^40^ ^]^ While this in vitro study highlights how the presence of mucins and mucin‐like glycoproteins can affect the lubrication properties of cellular surfaces, the effectiveness of rh‐lubricin at restoring the lubrication function of ocular surfaces that exhibit other DED phenotypes should be investigated further, along with alternative mechanisms of action on these ocular surfaces.

Together, the CAH and stress relaxation measurements at the cell‐cell interface illustrated that both surface roughness and surface chemistry contribute to the lubrication mechanism at the in vitro model ocular surface. We hypothesize that the mucin‐rich glycocalyx of the ocular epithelial cells promotes tear film infusion by establishing a negatively charged, hydrophilic surface, and the surface roughness of mature epithelial layers enhances liquid retention. Mucin‐like glycoproteins reduce friction at cellular interfaces during blink cycles through steric hindrance and charge repulsion. The model healthy and mucin‐deficient cell surfaces employed in these studies enabled well‐controlled mechanistic investigations of multiple shear adhesive interactions that occur at the ocular surface, shedding light on poorly understood interfacial properties and their influence on ocular surface biolubrication and hydration. In addition to furthering understanding of ocular surface biology, this biomimetic platform is anticipated to be useful in identifying design rules for ocular lubricants to supplement the damaged glycocalyx and interrogating lubricant‐ocular surface interactions.

## 4 Conclusion

In summary, we have shown that endogenous membrane‐tethered mucins differentially influence the adhesive properties of ocular epithelial cells, with minimal change in CAH observed on mucin‐deficient cell surfaces but significant increases in friction seen at cell‐cell interfaces when membrane‐tethered mucins are partially removed. Recombinant lubricin adsorbed on model ocular interfaces reduced the relaxation modulus at mucin‐deficient corneal and conjunctival epithelial cell interfaces, indicating that adsorbed mucin‐like glycoproteins can mitigate the loss in lubrication function induced by a deficiency in endogenous membrane‐tethered mucins. Concisely, a rough substrate and a hydrophilic surface chemistry synergistically improve lubrication performance at the model ocular surface during simulated blinks. Together, these results imply the potential of our biomimetic ocular surface model system with controlled mucin presentation in fundamental investigations of the interfacial properties of healthy and diseased ocular surface tissue.

## 5 Experimental Section

### Cell Culture

hTCEpi cells were generously donated by Professor Suzanne M.J. Fleiszig (University of California, Berkeley). HCjE cells were generously donated by Professor Ilene Gipson (Harvard Medical School) to the Byers Eye Institute at Stanford and obtained with the help of Professor Albert Wu's laboratory. hTCEpi cells were used between passages 56 and 70. HCjE cells were used between passages 6 and 20. hTCEpi and HCjE cells were cultured in the growth medium (GM) composed of Epilife supplemented with Human Corneal Growth Supplements (HCGS) and 1% penicillin‐streptomycin at 37 ^o^C and 5% CO_2_. To induce differentiation and stratification, the GM was replaced by the stratification medium containing Dulbecco modified Eagle/F12 medium, 10% fetal bovine serum, 10 ng mL^−1^ EGF, and 1% penicillin‐streptomycin.

### Immunofluorescent Imaging

For MUC1 immunofluorescent imaging, cells were cooled to 4 ^o^C, incubated with mouse anti‐human MUC1 antibodies (214D4) in culture medium (1:1000) for 2 h at 4 ^o^C and then incubated with Alexa fluorophore conjugated goat anti‐mouse IgG in culture medium (1:1000) for 45 min at 4 ^o^C. Cells were washed with PBS three times and fixed with 4% paraformaldehyde in PBS for 15 min. To label the O‐glycans on the surface of stratified cell cultures, the cells were incubated with fluorescein‐labeled Jacalin (1:1000) for 20 min. Hoechst 33 342 and CellMask Deep Red plasma membrane stain were used for nuclear and membrane staining according to the manufacturer's instructions. The cells were examined using a laser scanning confocal microscope (Zeiss LSM780) with a 63X water‐immersion objective.

### StcE Treatment

Stock solutions of purified StcE (1.65 mg mL^−1^) were generously supplied by Dr. Kayvon Pedram from Professor Carolyn Bertozzi's laboratory (Stanford University). A desired amount of StcE was added into the cell culture medium to evaluate mucin‐deficiency in hTCEpi and HCjE cultures. The working concentration of StcE ranged from 0.05 to 5 µg mL^−1^ in the culture medium.

### Contact Angle Hysteresis

An extended method on CAH measurement can be found in Supporting Materials. In brief, the advancing and receding CAs on hTCEpi epithelium were measured with a Ramé‐Hart CA goniometer using a captive bubble method. The CA was quantified by the CA plug‐in in the ImageJ software. The advancing and the receding CA were quantified by the DROPimage Advanced software offered by ramé‐hart. The CAH is defined as,

(2) $$\mathrm{CAH}=\cos\left(\theta_{\mathrm{receding}}\right)-\cos\left(\theta_{\mathrm{advancing}}\right)$$

### Surface Roughness Measurements

Cells were cultured in Lab‐tek II #1.5 glass bottom 8‐well chambers. The lipid bilayers were stained with CellMask Deep Red Plasma membrane stain and imaged with a Zeiss LSM780, 20X dry objective. A square field of view with a side length of 400 µm was chosen to analyze the surface roughness. The 3D Canny algorithm was used to detect the surface of the lipid bilayers. A customized MATLAB code was used to reconstruct the height map. The root‐mean‐square surface roughness was calculated according to the following equations,

(3) $$\mathrm{RM}S^{2}=\frac{1}{A}\int_{0}^{l_{y}}\int_{0}^{l_{x}}{\left(z\left(x,y\right)-m\right)}^{2}dx\;dy$$

where *m* is defined as,

(4) $$m\;=\frac{1}{A}\int_{0}^{l_{y}}\int_{0}^{l_{x}}z\left(x,y\right)dx\;dy$$

where *A* is the area for the field of view, *l_x_* and *l_y_* are the side lengths of the field of view, *z*(*x*, *y*) is the height profile as a function of two coordinates, and *m* is the mean profile height.

### Live Cell Rheometer (LCR)

An extended method on LCR can be found in Supporting Materials. In brief, a customized rheometer that resembles a sliding‐plate rheometer was used to measure the stress relaxation behavior of the cell layers. The apparent modulus as a function of time was defined as shear stress over shear strain and calculated through the following equation,

(5) $$G_{\mathrm{app}}\;\left(\gamma ,t\right)=\frac{\tau \left(t\right)}{\gamma }\;=\frac{F\left(t\right)\cdot d_{\mathrm{gap}}}{A\cdot d}$$

where *F(t)* is the force response monitored by a force sensor as a function of time, *A* is the area of the cell layer, *d* is the step movement applied by the micromanipulator, and *d*_gap_ is the gap height determined between the top and bottom plates.

### Live Cell Lubricin Adsorption Assay

Cy5‐conjugated recombinant lubricin (lubricin‐Cy5) samples were prepared with Antibody conjugation kits (Abcam) according to the manufacturer's instructions. StcE‐treated and non‐treated stratified hTCEpi (day 7) cells were incubated with lubricin‐Cy5 at 50 µg mL^−1^ for 2 h at 37 °C in growth CO_2_ independent medium. Negative controls were incubated with streptavidin‐Cy5 and positive controls were incubated with biotin‐PNA and streptavidin‐Cy5 under the same conditions. Nuclear counterstaining was done with Hoechst 33 342. The adsorption behaviors of lubricin‐Cy5 were visualized using a Zeiss LSM780 with a 20X dry objective. Z‐slices were taken before and after vigorous PBS washes. After live cell imaging, the lubricin‐Cy5 adsorbed cell layers were then fixed with 4% paraformaldehyde for 15 min and blocked with 5% BSA in PBS overnight at 4 °C. To verify the presence of lubricin molecules on cell surfaces, the cell layers were incubated with lubricin primary antibodies for 2 h and Alexa‐488 conjugated secondary antibodies for an hour before imaging.

### Statistical Analysis

Detailed descriptions of pre‐processing on apparent modulus from LCR can be found in Supporting Experimental Methods and previous literature.^[^ ^35^ ^]^ Data are reported in the format of mean ± standard error unless otherwise stated. Sample sizes are reported in respective figure legends and represent the numbers of biological replicates. If model assumptions were met, then data were analyzed by one‐way analysis of variance (ANOVA) with *α* = 0.05. The two‐tail *p*‐values were calculated by Student's *t*‐tests using Microsoft Excel. The number of biological replicates for each experiment condition was reported in the figures.

## Conflict of Interest

Professor Fuller received a research grant from Novartis Pharma AG.

## Author Contributions

C.L. and A.C.M. contributed equally to this work. All authors designed research; C.L. and A.C.M performed research; C.L., A.C.M, D.M., and G.G.F analyzed data; C.L., A.C.M, D.C.‐S., D.M., and G.G.F wrote the paper.

## Supporting information

Supporting Information

## Data Availability

The data that support the findings of this study are available from the corresponding author upon reasonable request.
