# Supplementary material for: Mucin‐Like Glycoproteins Modulate Interfacial Properties of a Mimetic Ocular Epithelial Surface
Source: Adv Sci (Weinh). 2021 Jun 29;8(16):2100841. doi: 10.1002/advs.202100841 (PMC8373091; doi:10.1002/advs.202100841)
Supplement: Supplementary file 1 — Supporting Information [file ADVS-8-2100841-s001.pdf]

© 2021 Wiley-VCH GmbH

ADVANCED SCIENCE
Open Access

Supporting Information

for *Adv. Sci.*, DOI: 10.1002/advs.202100841

Mucin-like Glycoproteins Modulate Interfacial Properties of a Mimetic Ocular Epithelial Surface

*Chunzi Liu, Amy C. Madl, Daniel Cirera-Salinas, Wolfgang Kress, Frank Straube, David Myung, Gerald G. Fuller**

Supporting Information

Mucin-like Glycoproteins Modulate Interfacial Properties of a Mimetic Ocular Epithelial Surface

*Chunzi Liu, Amy C. Madl, Daniel Cirera-Salinas, Wolfgang Kress, Frank Straube, David Myung, Gerald G. Fuller**

C. Liu, Dr. A. C. Madl, Dr. D. Myung, Prof. G. G. Fuller
Department of Chemical Engineering, Stanford University, Stanford, CA 94305 USA
E-mail: ggf@stanford.edu

Dr. D. Cirera-Salinas, Dr. W. Kress, Dr. F. Straube
Novartis Pharma AG, 4002, Basel, Switzerland

Dr. D. Myung
Department of Ophthalmology, Stanford University, Stanford, CA 94305 USA

Keywords: Ocular Surfaces, Dry Eye Disease, Lubricin, Muco-adhesion, Contact Angle Hysteresis, Interfacial Properties

1. Supplementary Experimental Methods

Contact angle hysteresis The advancing and receding contact angles on hTCEpi epithelium were measured with a Ramé-Hart contact angle goniometer using a captive bubble method.

Sample chamber A customized experiment chamber was assembled with diamond-cut glass slides. A 3D-printed inset was glued in the glass chamber. The indents on the inset was fitted to an 18*18 mm square coverslip.

Cell culture Square coverslips were sonicated in 1M HCl for 1 hour and rinsed in MilliQ-water for 10 times. Cleaned coverslips were stored in ethanol. Prior to cell seeding, the coverslips were allowed to air dry in a laminar hood. Cells were seeded on the coverslips and followed by the stratification process described in cell culture section if necessary. To induce morphological changes in hTCEpi cell layers, 1mM ethylene glycol-bis(β-aminoethyl ether)-N,N,N',N'-tetraacetic acid (EGTA) was supplemented to the culture medium. Cells were exposed EGTA-supplemented culture medium for 1 hour prior to the experiments.

Experiment procedure An inverted needle (Gauge #25) was attached to the air dispenser. The experimental chamber was fixed onto the goniometer tilting stage by tape. The needle and the chamber were plasma treated by a Diener Pico oxygen-plasma cleaner for 5 minutes prior to the experiments. The experimental chamber was filled with PBS immediately after the plasma treatment. The coverslips with cell culture were gently washed with PBS for three times before being loaded onto the 3D-printed inset with the cell side facing down. The surface tension of the chamber liquid was measured before each experiment with the pendant drop method to ensure that the PBS was not contaminated. During an experiment, an air bubble of 5 μL was created and freely attached against the cell surface. A video of the air bubble was recorded with the DROPimage Advanced software while the stage was tilting at 0.5 degrees/second. The chamber was cleaned with PBS after each experiment.

Data analysis The contact angle was quantified by the Contact Angle plug-in in the ImageJ software. The advancing and the receding contact angle were quantified by the DROPimage Advanced software offered by ramé-hart. The contact angle hysteresis is defined as shown in Equation 2.

AFM Single Cell Young's Modulus Single cell mechanics was quantified using a Bruker BioScope Resolve BioAFM in PeakForce QNM mode and a PFQNM-LC-ACAL cantilever

(Bruker, Santa Barbara, CA). The cells were cultured on 50-mm glass bottom petri dishes pretreated with collagen. A force volume of 10*10 μm (4*4 pixels) was acquired for each cell. The Hertzian contact model was used to extract the Young's modulus from the force curves by the NanoScope Analysis 1.9 software. The AFM was mounted on top of a Zeiss Axio Observer Z1 inverted epifluorescence microscope outfitted with a 20X objective.

Live Cell Rheometer (LCR) The basic design of the LCR has been described previously. [33-35] Some modifications have been implemented to improve the throughput of the instrument.

<u>Sample plate preparation</u> To prepare the bottom plate, a 3-mm deep opening was created by a ranch on the side of a 35-mm glass-bottom petri dish (Cellvis). To assemble the top plate, a 12-mm circle coverslip was glued with Norland NOA60 optical adhesives onto a 3D-printed handler for the force sensor to approach. The handlers were printed either by an Ultimaker 2 with polylactic-acid or through Shapeways 3D printing service with versatile plastics.

<u>Cell culture</u> Bottom and top plates were coated with rat-tail Type I collagen at 5 $\mu g/cm^2$ for 30 minutes at 37°C. hTCEpi cells were seeded onto the bottom plates at 350,000 cells/ml and HCjE were seeded onto the top plates at 200,000 cells/ml in the growth medium (GM). The top plates were kept in 24-well plastic tissue culture plate with the coverslip side facing up. After the cells reached confluence, the GM was replaced with the stratification medium. LCR experiments were done on the seventh day of stratification. To generate mucin-deficient dry-eye model, cells were treated with 0.5 μg/ml StcE in stratification medium overnight.

<u>Adhesion assay</u> On the day of the experiment, the cell culture was washed with PBS three times, and switched to CO_2-independent medium supplemented with L-glutamine, 10% fetal bovine serum, 10 ng/mL EGF, and 1% penicillin-streptomycin. To test the lubrication effect of lubricin, stock solutions of lubricin (2.20 mg/mL amino acid concentration, 4.40 mg/mL total protein concentration) were added into full CO_2-independent medium to a desired final concentration. As control groups, bovine serum albumin was reconstituted into 5 mg/ml stock solution in PBS and then added into full CO_2-independent medium to a desired final concentration.

To start the adhesion assay, the top plates were gently placed onto the bottom plates. The assembled plates were kept at 37°C and ambient CO_2 level for 2 hours before the step strain experiments.

Step strain experiment The experiment was controlled by a customized MATLAB code. After the force sensor was brought in contact with the top plate, a user-defined step motion was applied through the micromanipulator. A DAQ board (DAQ USB6008, National Instruments) collected the voltage readings as a function of time from the force sensor which were converted to force levels, $F(t)$, using a known conversion factor given by the manufacturer. The nominal contact area, A, was determined as the area of a 12-mm circle coverslip. The shear stress exerted on the corneal epithelial cell monolayers was defined as shear force over area, or $F(t)/A$. The gap, d_{gap}, was determined in gap height measurement (see below). The shear strain exerted on the corneal epithelial cell monolayer was defined as the shear distance over the gap height in the normal direction, or d/d_{gap}.

The apparent modulus as a function of time was defined as shear stress over shear strain and calculated through Equation 5, where $\tau(t)$ is the shear stress as a function of time, γ is the shear strain, and d is the step movement applied by the micromanipulator. At least two step strain experiments (technical replicates) were performed on each sample. The average of technical replicates was reported as a biological replicate.

Data Analysis One-component, two-component, three-component exponentials have been applied to fit the relaxation curves. The equation for the two-component exponentials is shown in Eq 1. An F-test on the variance of sum of squared residuals has shown that the two-component exponential function performed statistically better than the one-component exponential function, but was not outperformed by the three-component exponential function.

The peak modulus was defined as the first modulus value after the step strain was applied. The plateau modulus was extracted from the two-component exponential fitting, G_{20}. Statistical analysis was performed using a two-tail Welch's t-test.

Gap height measurement Cell cultures on top and bottom plates were incubated with Hoechst 33342 (1:1000) for 20 minutes at 37°C, and then sample plates were assembled as previously described in the adhesion assay. Confocal z-stacks in five fields of view were acquired with Zeiss LSM 780 with a 20X air objective for each sample. Gap height was arbitrarily determined as height difference between 10% of maximum fluorescent intensity. The results for gap height measurements are shown in Fig. S3.

Alternatively, top and bottom plates were coated with fluorescent beads. To coat coverslips with fluorescent beads, stock solutions of RFP colloidal beads were diluted into 0.005% (w/v)

working solutions with ethanol. Then 0.2 mL and 0.4 mL of working solutions were added to top and bottom plates, respectively, and allowed to air dry for 30 minutes in laminar hoods. Sample plate preparation and imaging were proceeded as previously described. The gap height was defined as the difference between the beads plane.

Characterization of lubricin samples Concentration of recombinant human lubricin stock solution was determined by Bradford assay according to manufacturer's instruction.

Western blot Protein solutions were denatured at 95°C for 5 minutes in loading buffer (16% 2-Mercaptoethanol, 200mM Tris-HCl pH 6.9, 8% SDS, 40% glycerol, 400 mM DTT, 0.4% bromophenol blue). The samples were separated on NuPAGE 3-8% Tris-acetate gels and transferred to nitrocellulose membranes (Amershan Protran 0.45 um NC, GE Healthcare) for 2 hours at 100 V (14). Membranes were blocked with 5% milk in phosphate buffered saline + 0.1% Tween 20 (PBST) for 2 hours. Primary antibodies (9G3) and lectins were diluted to 1:1000 in 5% milk PBST and incubated on membranes overnight at 4°C. HRP-conjugated or fluorescently labeled secondary antibodies were diluted to 1:10000 in 5% milk PBST and incubated for an hour at room temperature prior to the chemiluminescence reaction or fluorescent imaging.

Dynamic light scattering The dynamic light scattering was performed on a Brookhaven Instrument Nanobrook Omni. Stock solutions were centrifuged at 5000 rpm for 5 minutes prior to the experiments to remove potential aggregates. Samples were placed in a 15-ul cuvette for the measurements.

2. Supplementary Figures

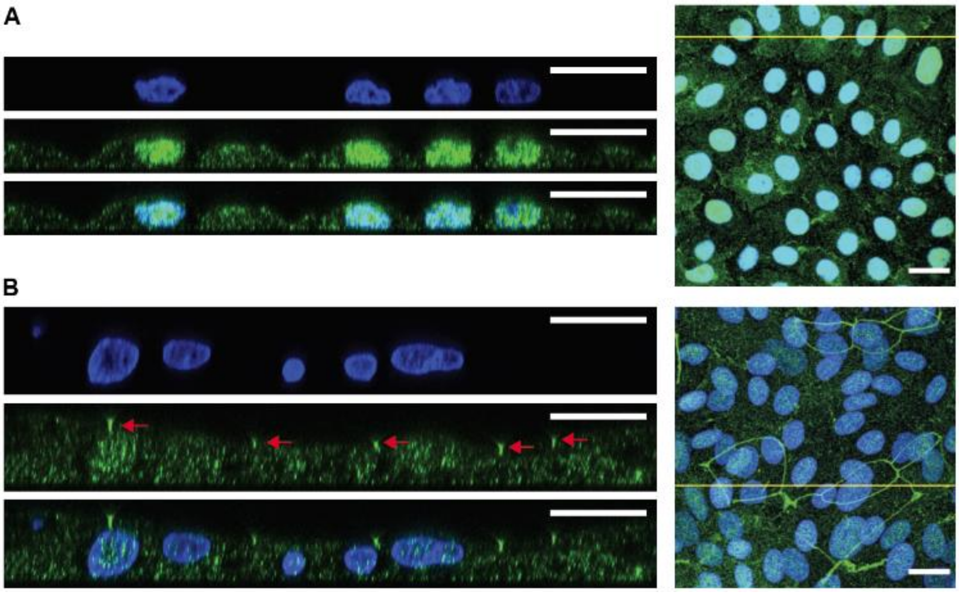

Figure S1. The localization of zonula occludens-1 (ZO-1) in hTCEpi over differentiation process. The localization changed from (**A**) the nucleus in monolayers to (**B**) the tight junctions after the differentiation and stratification process. Left: Cross sections of the cell layers at the positions indicated by the yellow lines. The tight junctions are indicated by the red arrows. Right: Maximum-intensity projections. Blue: Nuclear staining. Green: ZO-1. Scale bar: 20 µm.

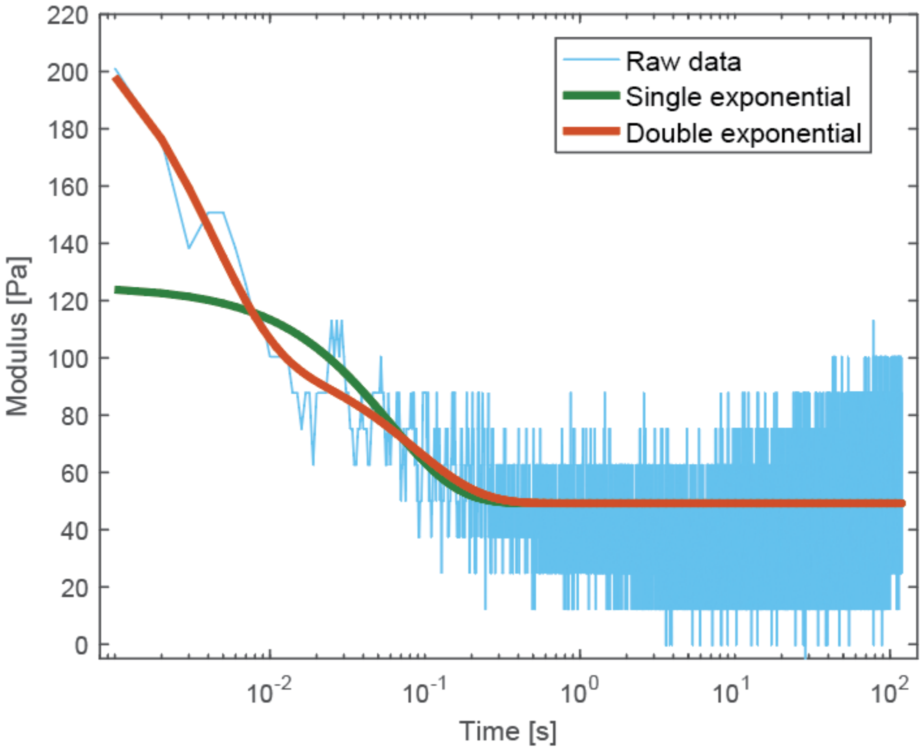

Figure S2. The goodness of fit (GoF) for a representative stress relaxation curve using a single exponential model and a double exponential model. The double exponential model captured the fast relaxation behavior in the first two decades of the relaxation curves in contrast to the single exponential fit.

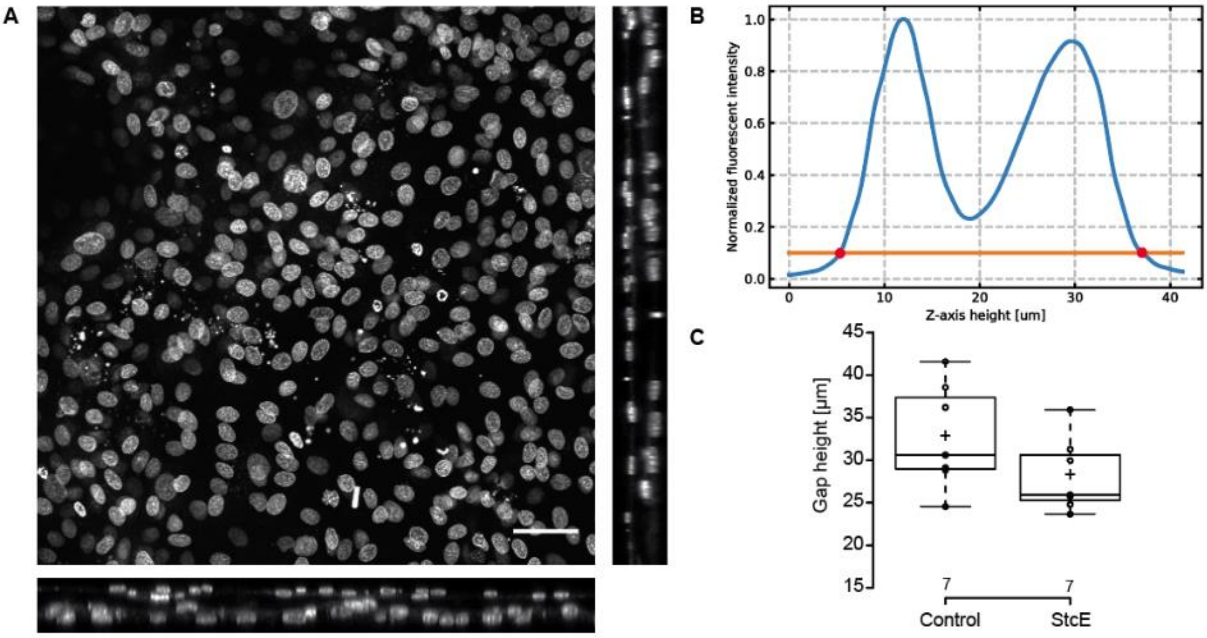

Figure S3. Gap height measurements for LCR with live cell fluorescent imaging. (**A**) Z-projects of stratified hTCEpi/HCjE cell layers with Hoechst staining. Scale bar: 50 µm. (**B**) Normalized fluorescent intensity averaged across x-y plane as a function of z-axis heights. The height of the hTCEpi/HCjE cell layer was defined as the difference between the intercepts (red dots) of the normalized fluorescent intensity and 10% maximum intensity (orange line). (**C**) Gap height between control and StcE-treated cell layers.

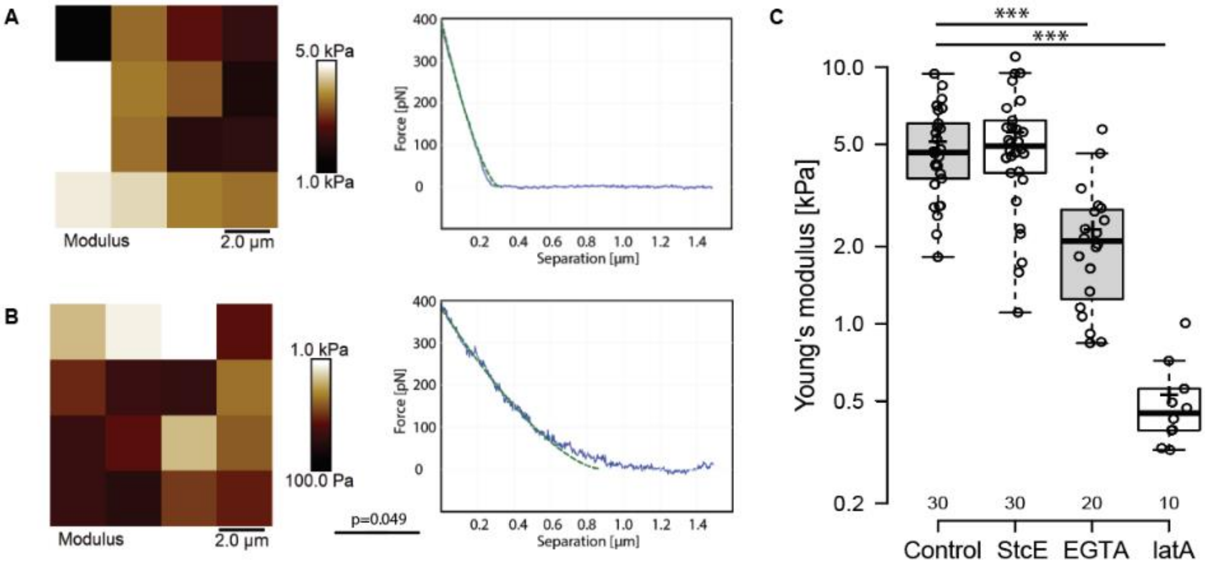

Figure S4. Atomic force microscopy measurements on the Young's modulus of hTCEpi single cells. Representative force-volume (FV) graphs and force curves for (**A**) control hTCEpi single cells and (**B**) latA-treated hTCEpi single cells from atomic force microscopy. The Young's modulus was calculated by fitting the force curve (blue) to a Hertzian contact model (green). (**B**) Young's moduli of stratified hTCEpi cells under StcE treatment and EGTA treatment. Student t-test was used to report the two-tail *p*-value. ***: $p \leq 0.001$.

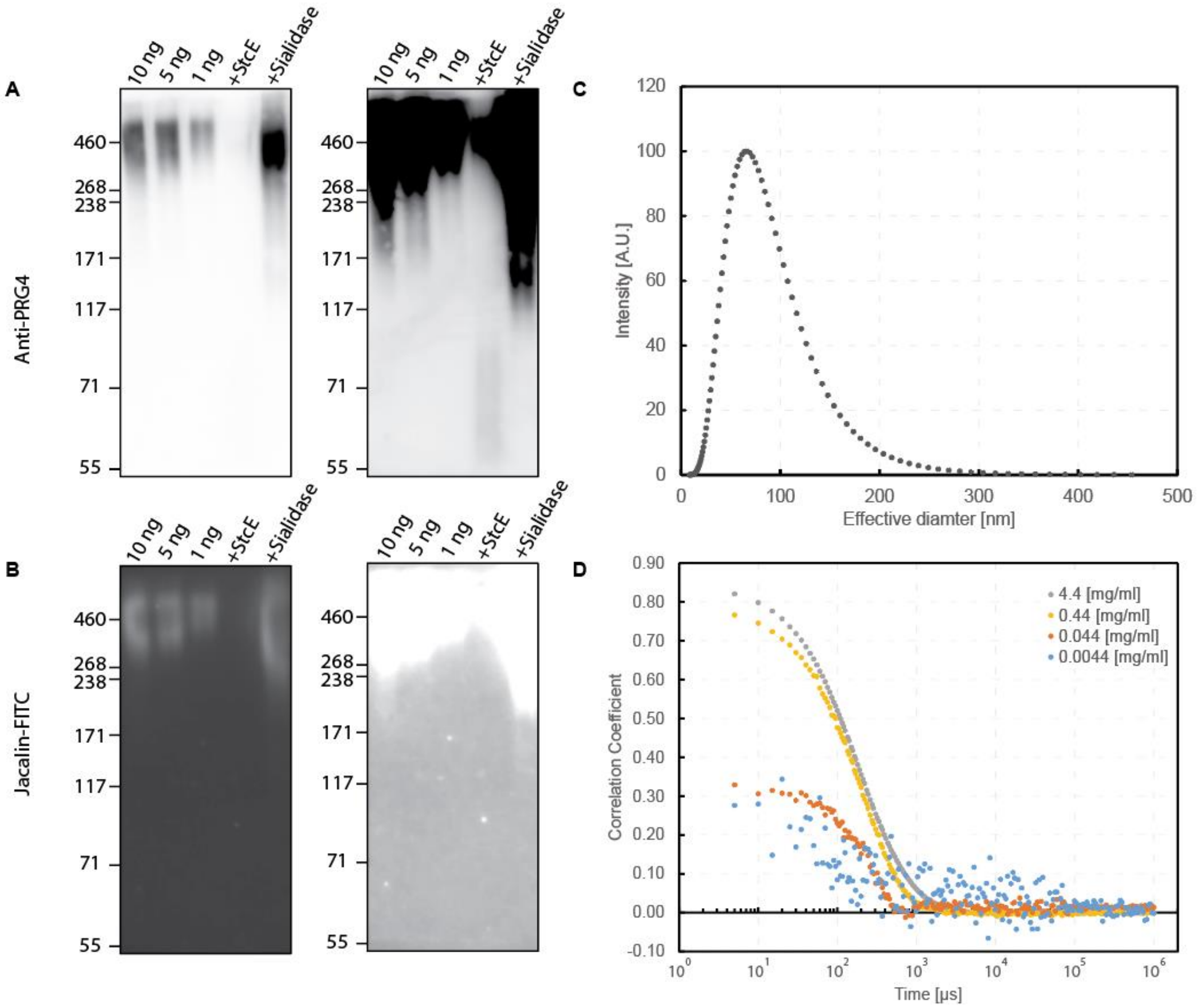

Figure S5. Characterizations of recombinant human lubricin (rh-lubricin). (**A**) Western blot and (**B**) lectin blot of rh-lubricin under short exposure time (left) and long exposure time (right). After StcE treatment, a low molecular weight band (~60 kDa) showed in Western blot but not in lectin blot under long exposure time. (**C**) Effective diameter of rh-lubricin measured from dynamic light scattering (DLS). (**D**) Representation correlation coefficient measurements from DLS at different concentrations of rh-lubricin.

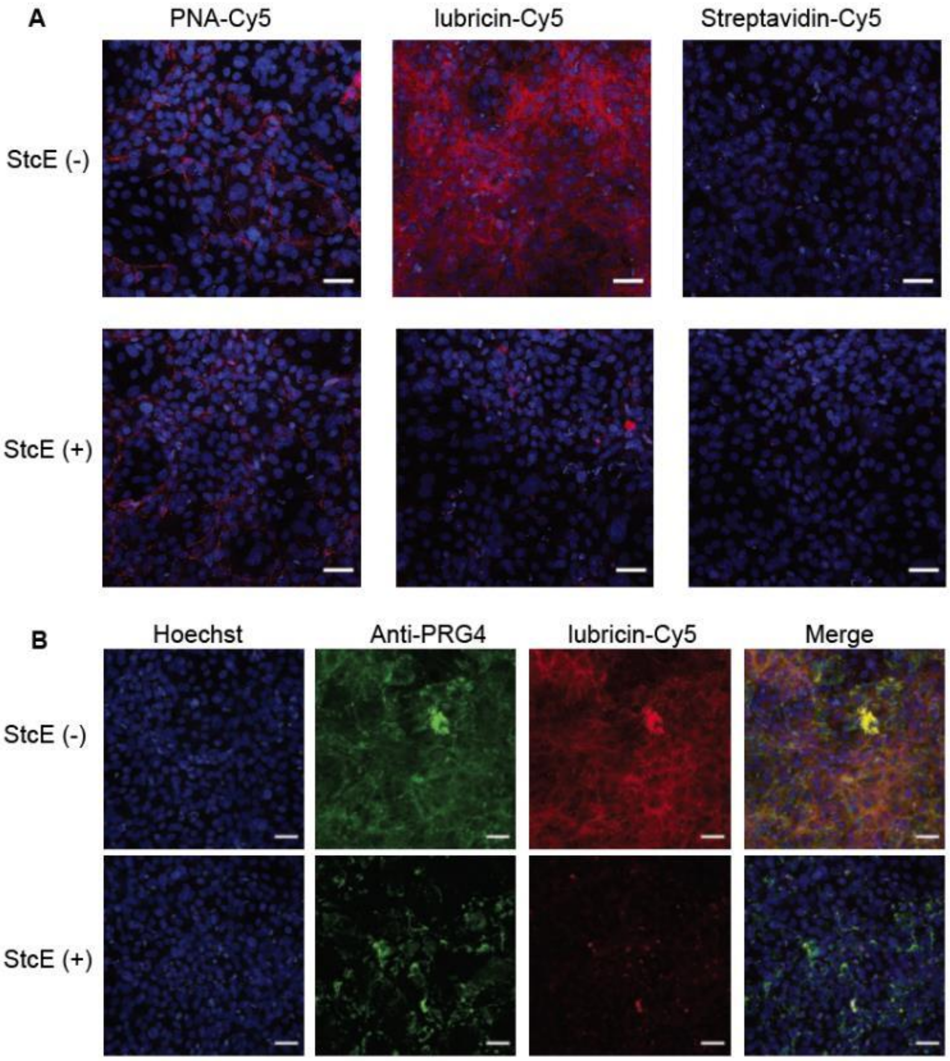

Figure S6. Adsorption behaviors of rh-lubricin on differentiated hTCEpi cell surfaces. (**A**) Adsorption behavior of Cy5-conjugated recombinant lubricin molecules on stratified hTCEpi surfaces with StcE treatment. Cy5-conjugated Peanut agglutinin (PNA) probes the core-1 glycans which are prevalently present in glycosylated membrane proteins and are intact to the mucin-specific StcE treatment. Low signal in the streptavidin-Cy5 channel indicates low levels of non-specific interactions between Cy5 and the cell surface. (**B**) Immunofluorescence imaging confirmed the presence of lubricin on stratified hTCEpi cell layers after the adsorption of Cy5-conjugated recombinant lubricin. Scale bar: 50 μm.

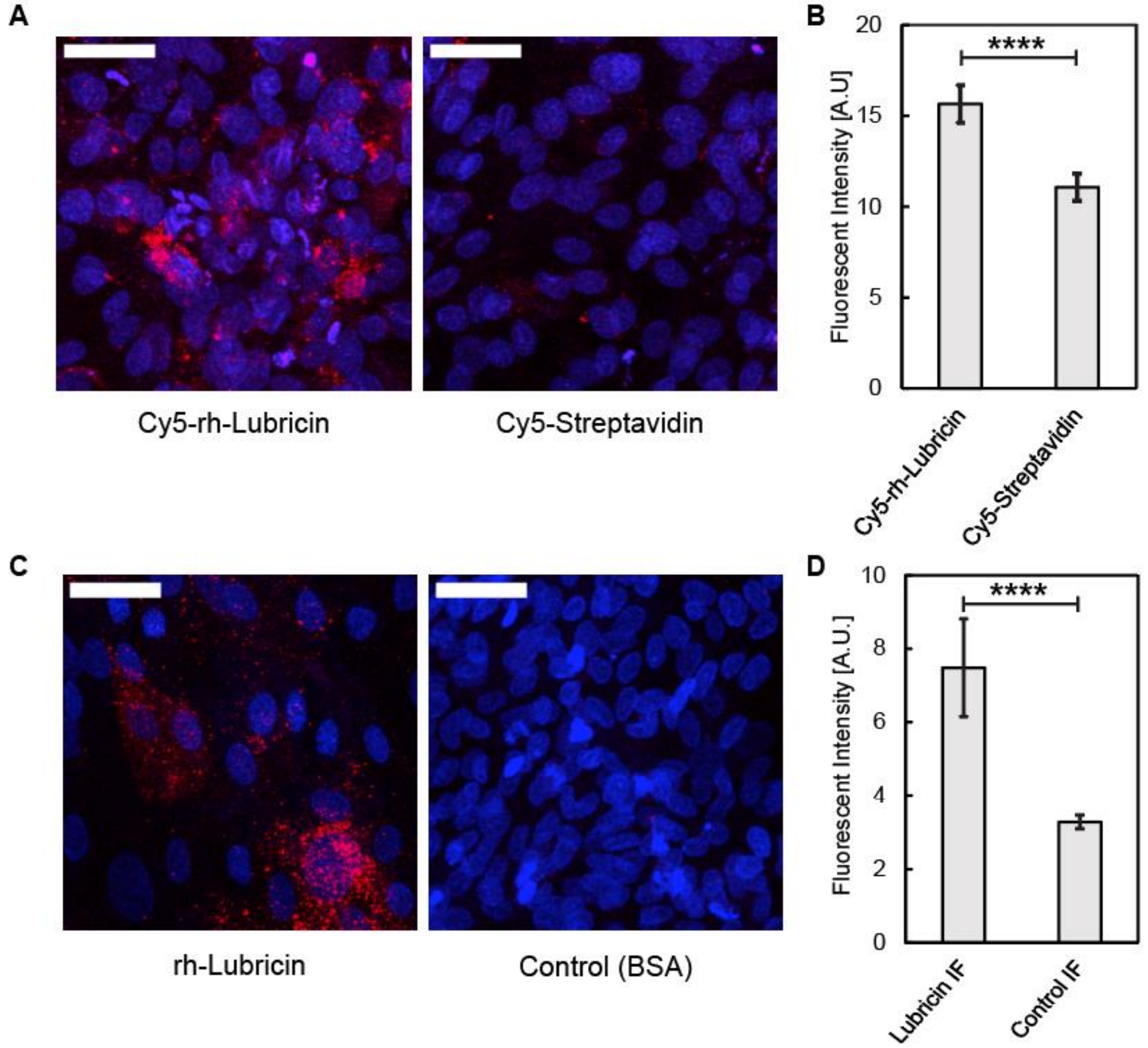

Figure S7. Quantifications of rh-lubricin adsorption on StcE-treated hTCEpi cell surfaces. (**A**) Representative live cell images of StcE-treated hTCEpi cell surfaces after the adsorption of Cy5-rh-lubricin and Cy5-streptavidin. The contrasts were normalized to the level of Cy5-streptavidin. Blue: nucleus. Red: Cy5. Scale bar: 40 µm. (**B**) Quantification of Cy5 fluorescent intensities after adsorptions of rh-lubricin and streptavidin. (**C**) Representative immunofluorescent images of StcE-treated hTCEpi cell surfaces after the adsorption of rh-lubricin and bovine serum albumin (BSA). Blue: nucleus. Red: rh-lubricin. Scale bar: 40 µm. (**D**) Quantification of Alexa-555 fluorescent intensities after adsorptions of rh-lubricin and BSA. The mean intensity was calculated by Zen Blue over five (B) and ten (D) fields of view of 425 µm × 425 µm. The error bar represents standard deviation. The significance was analyzed with two-tail Student's t-test. ****: $p \leq 0.0001$

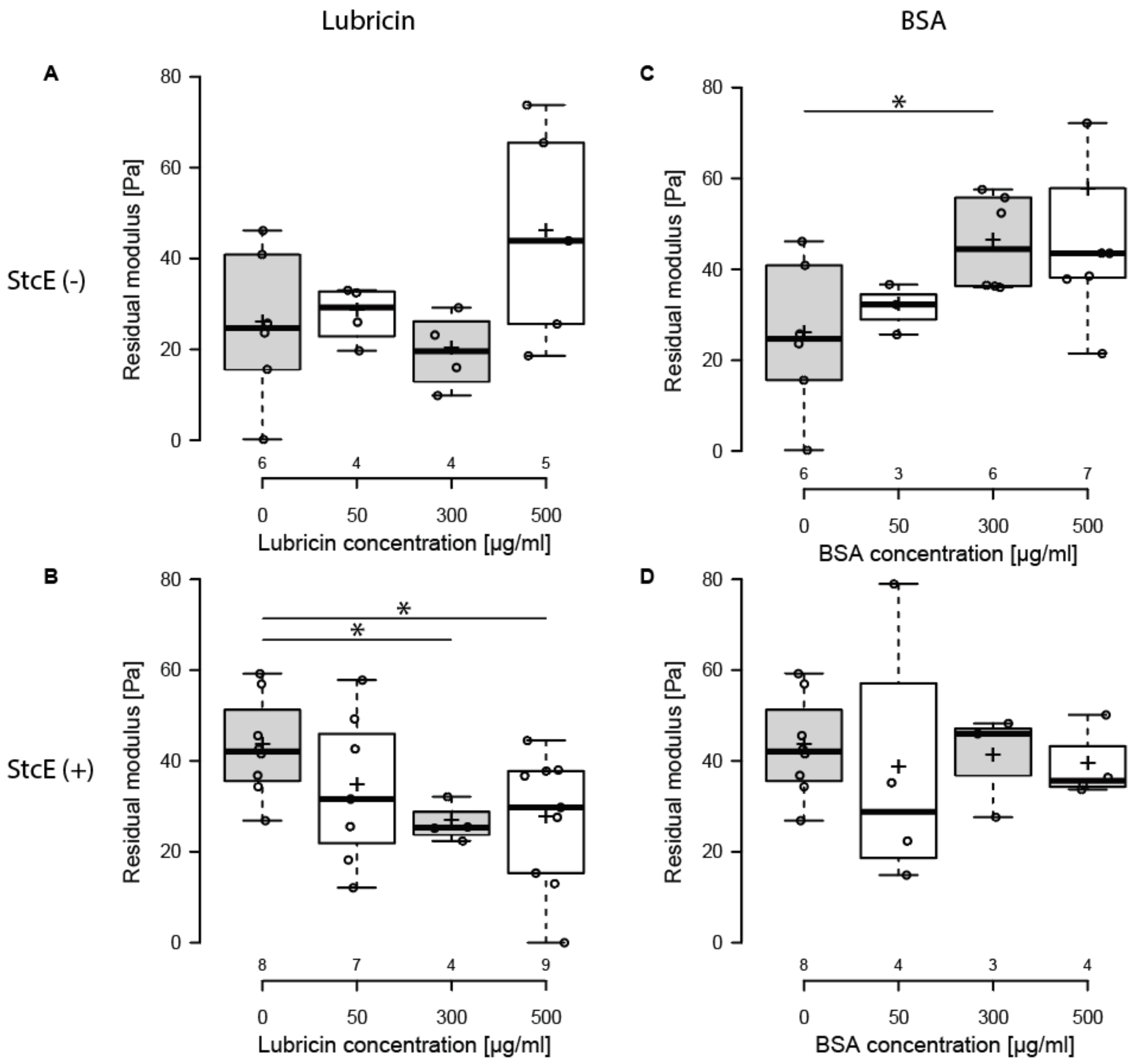

Figure S8. Residual moduli for stratified hTCEpi/HCjE models at different concentrations of supplemented proteins. (**A**, **B**) Control/StcE treated model supplemented with lubricin. (**C**, **D**) Control/StcE treated model supplemented with BSA. Student‘s t-test was used to report the two-tail *p*-value. *: $p<0.05$.
